# Comorbid Conditions and Complications in Body Contouring Surgery: A Retrospective Review

**DOI:** 10.1093/asjof/ojad080

**Published:** 2023-08-31

**Authors:** Kassra Garoosi, Leela Mundra, Kayvon Jabbari, Julian Winocour, Matthew L Iorio, David W Mathes, Christodoulos Kaoutzanis

## Abstract

**Background:**

Body contouring procedures have significantly increased in popularity in the United States.

**Objectives:**

The authors sought to understand, categorize, and classify patients’ experiences with postoperative complications following common body contouring procedures.

**Methods:**

PearlDiver (PearlDiver Technologies, Colorado Springs, CO), a database with over 90 million patients, was queried to identify patients who had undergone body contouring procedures between 2010 and 2021 using current procedural terminology (CPT) codes. The authors identified patients who underwent panniculectomy, abdominoplasty, brachioplasty, thighplasty, mastopexy, breast augmentation, augmentation mastopexy, breast reduction, and liposuction for analysis. They reviewed combined procedures and analyzed risk factors associated with the most common complications.

**Results:**

There were 243,886 patients included in the study. The majority of patients were female, between 50 and 59 years old, and had their procedures performed in the southern United States. There were an average of 25,352 procedures per year. The majority of cases involved breast surgeries. The most common preoperative comorbid conditions diagnosed 1 year before surgery were hypertension, obesity, and diabetes. The most common postoperative complications within 90 days were wound dehiscence, hematoma, and urinary tract infection. A logistic regression evaluating the association of the preoperative comorbid conditions with postoperative complications found that patients with obesity, tobacco use, diabetes, and hypertension had an increased risk of developing wound dehiscence, hematoma, and surgical-site infection.

**Conclusions:**

The data suggest that patients with obesity, tobacco use, diabetes, and hypertension undergoing body contouring surgery are at greater risk of developing wound dehiscence, hematomas, and surgical-site infections. Understanding this data is imperative for providers to adequately identify associated risk factors, stratify patients, and provide adequate perioperative counseling.

**Level of Evidence: 2:**

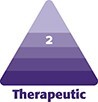

Body contouring procedures are becoming increasingly popular. From 2000 to 2020, there was a 65% increase in mastopexy, a 3974% increase in the number of lower body lifts, and a 4174% increase in the number of patients undergoing upper arm lifts.^1^ The rising popularity is partially due to a greater acceptance of plastic surgery in the community, desire for a more youthful appearance, as well as rising popularity of bariatric surgery. After massive weight loss, patients complain of excessive skin or soft tissue that can cause irritation, rashes and infection within intertriginous areas, or difficulty with daily activities such as ambulation or hygiene.

Prior studies have reported various postoperative complications after body contouring surgery, including wound complications such as dehiscence and delayed wound healing, seroma, hematoma, infection, venous thromboembolism, and nerve injuries.^2-4^ Other authors have found patient-risk factors, such as obesity and male patients, to be associated with postoperative complications.^5-8^ Despite this available data, body contouring surgeries continue to increase in frequency, and there is a relative paucity of data as it relates to the impact of preoperative comorbid conditions on postoperative complications.

The objective of this study was to assess the incidence of common preoperative comorbidities and postoperative complications in patients undergoing body contouring procedures. Furthermore, we attempted to analyze the relationship between comorbid conditions with the development of postoperative complications following body contouring surgery.

## METHODS

The study used the PearlDiver patient database of privately insured patients across all regions of the country. PearlDiver (PearlDiver Technologies, Colorado Springs, CO) is a commercially available online database encompassing 153 million unique patients from both the Humana Claims data set and the Medicare Standard Analytic File between January 1, 2010 and March 30, 2020. Services that do not provide their data to PearlDiver, or patients who do not have insurance coverage, were not included in the database. PearlDiver tracks patients across all episodes of care in which their insurance was utilized. The database utilizes International Classification of Disease, 9 Codes (ICD-9) and 10 Codes (ICD-10), or current procedural terminology (CPT) codes to associate all billable encounters. When compared with other commonly queried insurance databases, PearlDiver provides a large sample size covering many regions of the country and includes patients from various types of practices.

The information was accessed through a secure server using PearlDiver's proprietary software. The study used deidentified patient data from the database; however, groups with 10 patients or fewer were not reported by the database to protect privacy. As this study contained only deidentified aggregate data, patient consent was not required and the Colorado Multiple Institutional Review Board designated it as nonhuman research not in need of approval. In order to ensure the validity of the study, we used the Strengthening the Reporting of Observational Studies in Epidemiology guidelines and checklist in performing the study.

### Study Cohort

The study included patients undergoing body contouring procedures. Body contouring procedures included panniculectomy, abdominoplasty, brachioplasty, thighplasty, mastopexy, breast augmentation, augmentation mastopexy, breast reduction, and liposuction. These procedures were identified by CPT codes. The codes included CPT-15830 for panniculectomy; CPT-15830 and CPT-15847 for abdominoplasty; CPT-15836 for brachioplasty; CPT-15832 for thighplasty; CPT-19316 for mastopexy; CPT-19325 for breast augmentation; CPT-19316 and CPT-19325 for augmentation mastopexy; CPT-19318 for breast reduction; and CPT-15877, CPT-15878, and CPT-15879 for trunk liposuction. Patients who had active insurance at least 1 year prior to and 90 days after the date of surgery were included. This ensured that all patients were accounted for and not lost to follow-up during the study period analyzed.

Comorbid conditions included in the study were chronic pulmonary disease, renal disease, cardiac disease, diabetes, hypertension, obesity, peripheral artery disease, and smoking. We verified that all patients were diagnosed with a respective comorbid condition 1 year prior to surgery.

Postoperative complications included in the study were wound dehiscence, hematoma formation, surgical-site infection, urinary tract infection, pneumonia, and venous thromboembolism. Complications were defined using their respective ICD-10 codes. Only complications that occurred within 90 days of the index procedure were included in the study. The 90-day period was selected based on commonly observed timing of complications postoperatively.

### Statistical Analysis

Baseline demographics were obtained for all patients undergoing body contouring procedures. We tabulated the frequency of the most common comorbid conditions and postoperative complications. A logistic regression was performed to evaluate how preoperative comorbid conditions impact the development of surgical-site postoperative complications, and a subgroup analysis examining preoperative comorbid conditions and postoperative outcomes among the individual procedures was performed. A *P*-value of ≤.05 was considered statistically significant.

## RESULTS

A total of 243,886 patients who underwent body contouring surgery and 290,592 counted body contouring procedures were observed in the 10-year study period (Table 1). The majority of patients were females (235,481; 96.6%), between 50 and 59 years old (65,355; 25.8%), and had their procedures performed in the Southern United States (111,921, 38.6%). There was an average of 25,352 procedures performed each year, an approximate equal distribution of cases from 2010 to 2020, although there was a decline in the number of cases performed in 2020 (Table 2).

**Table 1.** Body Contouring Procedures

Procedure	No. of patients	Percentage
Body contouring procedures	290,592	100
Abdomen		
Abdominoplasty	17,202	5.9
Panniculectomy	46,198	15.9
Breast		
Augmentation/mastopexy	3270	1.1
Breast augmentation	12,554	4.3
Breast reduction	149,925	51.6
Mastopexy	37,817	13.0
Extremities		
Brachioplasty	2710	0.9
Thighplasty	2206	0.8
Liposuction	18,710	6.4
Combined procedures	267,218	100
Abdomen procedures (abdominoplasty, panniculectomy, liposuction)	61,887	23.2
Breast procedures (breast augmentation, breast reduction, mastopexy)	191,524	71.7
Extremity procedures (brachioplasty, thighplasty)	4551	1.7
Abdomen + breast	7310	2.7
Abdomen + extremities	1319	0.5
Breast + extremities	486	0.2
Abdomen + breast + extremities	141	0.05

**Table 2.** Patient Demographics

Demographics	No. of patients	Percentage
Gender	243,886	100
Female	235,481	96.6
Male	8405	3.4
Age	253,589	100
<18	2885	1.1
18-29	30,032	11.8
30-39	38,417	15.1
40-49	59,022	23.3
50-59	65,355	25.8
60-69	43,875	17.3
70+	14,003	5.5
Year	278,875	100
2010	25,485	9.1
2011	25,520	9.2
2012	26,049	9.3
2013	26,945	9.7
2014	26,021	9.3
2015	25,511	9.1
2016	25,834	9.3
2017	24,249	8.7
2018	24,194	8.7
2019	26,348	9.4
2020	22,719	8.1
Region	289,521	100
Midwest	71,252	24.6
Northeast	71,611	24.7
South	111,921	38.7
West	33,571	11.6
Unknown	1166	0.4

Overall, breast procedures were the most common procedures performed (*n* = 203,566; 70.1%). The most common procedure performed was breast reduction (*n* = 149,925; 51.6%), followed by panniculectomy (*n* = 46,198; 15.9%), and mastopexy (*n* = 37,817; 13.0%; Table 1). We identified hypertension (73,091; 30.0%), obesity (55,051; 22.6%), and diabetes (34,794; 14.3%), as the most common preoperative comorbid conditions (Table 3). The most common postoperative complications within 90 days were wound dehiscence (7944; 3.3%), followed by hematoma (6224; 2.5%) and urinary tract infection (5765; 2.4%; Table 4).

**Table 3.** Comorbid Conditions Among Patients Undergoing Body Contouring Procedures

Conditions	Body contouring	Abdominoplasty	Panniculectomy	Augmentation/mastopexy	Breast augmentation	Breast reduction	Mastopexy	Brachioplasty	Thighplasty	Liposuction
No. of patients	243,886	17,202	46,198	3270	12,554	149,925	37,817	2710	2206	18,710
Obesity	55,051	6916	20,106	285	874	29,659	4867	1167	1055	3439
Smoking	28,555	2094	6249	472	1851	14,527	5891	389	335	2325
Cardiac disease	17,859	1309	4710	271	997	8123	3767	355	230	1433
Chronic pulmonary disease	31,536	2726	8067	377	1369	17,516	4590	547	469	2206
Diabetes	34,794	3761	12,552	357	1043	16,416	5062	752	519	2204
Hypertension	73,091	6104	18,863	802	2661	39,345	12,580	1253	948	5323
Peripheral vascular disease	5370	460	1665	67	229	2289	1016	148	90	490
Renal disease	5709	432	2057	55	196	2198	865	370	94	395

**Table 4.** Complication Rates Among Patients Undergoing Body Contouring Procedures

Complications	Body contouring	Abdominoplasty	Panniculectomy	Augmentation/mastopexy	Breast augmentation	Breast reduction	Mastopexy	Brachioplasty	Thighplasty	Liposuction
No. of patients	243,886	17,202	46,198	3270	12,554	149,925	37,817	2710	2206	18,710
Hematoma	6224	905	2616	41	147	2733	657	82	142	318
Surgical-site infection	4336	596	1973	26	76	1857	350	73	151	318
Wound dehiscence	7944	935	3097	54	141	3866	748	125	270	329
Urinary tract infection	5765	479	1730	71	264	2827	865	76	87	372
Pneumonia	1607	148	608	11	51	684	201	29	23	111
Venous thromboembolism	1379	173	591	0	35	561	175	14	20	117

A logistic regression analysis on all patients undergoing body contouring procedures was performed, investigating the relationship between preoperative comorbid conditions and surgical-site postoperative complications, and found that patients diagnosed with diabetes, hypertension, obesity, and tobacco use have greater odds of developing surgical-site infection, wound dehiscence, and hematoma formation (Table 5).

**Table 5.** Logistic Regression of Preoperative Comorbid Conditions and Postoperative Complications

Conditions	Obesity		Tobacco		Diabetes		Hypertension	
	OR (CI)	*P*-value	OR (CI)	*P*-value	OR (CI)	*P*-value	OR (CI)	*P*-value
Wound dehiscence								
Body contouring	1.89 (1.83-1.97)	<.01	1.71 (1.65-1.77)	<.01	1.50 (1.45-1.56)	<.01	1.60 (1.54-1.67)	<.01
Abdominoplasty	2.06 (1.74-2.44)	<.01	1.61 (1.46-1.78)	<.01	1.48 (1.33-1.65)	<.01	1.75 (1.54-1.99)	<.01
Panniculectomy	1.88 (1.71-2.08)	<.01	1.59 (1.50-1.68)	<.01	1.52 (1.43-1.62)	<.01	1.78 (1.65-1.93)	<.01
Augmentation/mastopexy	1.18 (0.849-1.62)	.322	1.49 (1.10-2.02)	<.01	0.861 (0.597-1.23)	.418	1.84 (1.33-2.57)	<.01
Breast augmentation	1.51 (1.26-1.79)	<.01	1.69 (1.44-1.98)	<.01	1.01 (0.828-1.22)	.929	1.76 (1.48-2.09)	<.01
Breast reduction	1.86 (1.76-1.97)	<.01	1.75 (1.67-1.84)	<.01	1.37 (1.30-1.44)	<.01	1.48 (1.40-1.56)	<.01
Mastopexy	1.58 (1.44-1.72)	<.01	1.59 (1.46-1.73)	<.01	1.27 (1.15-1.39)	<.01	1.23 (1.12-1.35)	<.01
Brachioplasty	2.47 (1.67-3.81)	<.01	1.61 (1.27-2.03)	<.01	1.32 (1.03-1.71)	.0312	1.56 (1.12-2.19)	<.01
Thighplasty	2.57 (1.79-3.78)	<.01	1.45 (1.17-1.79)	<.01	1.28 (1.02-1.60)	.0305	1.35 (1.03-1.78)	.0316
Liposuction	1.44 (1.28-1.62)	<.01	1.72 (1.54-1.92)	<.01	1.27 (1.12-1.43)	<.01	1.37 (1.22-1.55)	<.01
Hematoma								
Body contouring	1.20 (1.16-1.25)	<.01	1.38 (1.34-1.43)	<.01	1.39 (1.35-1.45)	<.01	1.72 (1.66-1.78)	<.01
Abdominoplasty	1.47 (1.27-1.71)	<.01	1.34 (1.22-1.48)	<.01	1.29 (1.17-1.44)	<.01	1.72 (1.52-1.95)	<.01
Panniculectomy	1.32 (1.21-1.45)	<.01	1.34 (1.27-1.42)	<.01	1.32 (1.24-1.40)	<.01	1.65 (1.53-1.78)	<.01
Augmentation/mastopexy	1.04 (0.790-1.36)	.779	1.40 (1.09-1.80)	<.01	1.28 (0.962-1.70)	.0867	1.59 (1.22-2.10)	<.01
Breast augmentation	1.21 (1.04-1.41)	.0111	1.26 (1.10-1.44)	<.01	1.29 (1.10-1.51)	<.01	1.67 (1.45-1.93)	<.01
Breast reduction	1.21 (1.15-1.28)	<.01	1.39 (1.33-1.47)	<.01	1.41 (1.34-1.49)	<.01	1.74 (1.65-1.84)	<.01
Mastopexy	1.11 (1.03-1.19)	<.01	1.32 (1.23-1.42)	<.01	1.14 (1.05-1.24)	<.01	1.37 (1.27-1.49)	<.01
Brachioplasty	1.44 (1.03-2.06)	.0391	1.19 (0.932-1.51)	.163	1.17 (0.906-1.51)	.236	1.52 (1.11-2.12)	.0115
Thighplasty	1.73 (1.22-2.54)	<.01	1.17 (0.925-1.48)	.187	1.22 (0.958-1.55)	.108	1.67 (1.23-2.28)	<.01
Liposuction	1.02 (0.920-1.13)	.731	1.22 (1.10-1.35)	<.01	1.07 (0.954-1.19)	.26	1.58 (1.42-1.76)	<.01
Surgical-site infection								
Body contouring	2.23 (2.12-3.35)	<.01	1.79 (1.71-1.86)	<.01	1.38 (1.33-1.45)	<.01	1.45 (1.38-1.52)	<.01
Abdominoplasty	2.16 (1.75-2.70)	<.01	1.57 (1.39-1.78)	<.01	1.30 (1.14-1.49)	<.01	1.79 (1.53-2.12)	<.01
Panniculectomy	2.06 (1.82-2.34)	<.01	1.70 (1.59-1.83)	<.01	1.40 (1.30-1.51)	<.01	1.63 (1.48-1.79)	<.01
Augmentation/mastopexy	1.83 (1.16-2.86)	<.01	1.55 (1.003-2.39)	.0463	0.721 (0.406-1.23)	.246	1.05 (0.663-1.66)	.836
Breast augmentation	2.37 (1.88-2.97)	<.01	1.44 (1.15-1.79)	<.01	1.04 (0.798-1.35)	.774	1.32 (1.04-1.67)	.022
Breast reduction	2.03 (1.89-2.19)	<.01	1.82 (1.71-1.94)	<.01	1.21 (1.13-1.29)	<.01	1.35 (1.25-1.45)	<.01
Mastopexy	1.95 (1.73-2.19)	<.01	1.58 (1.40-1.77)	<.01	0.943 (0.827-1.07)	.375	1.02 (0.901-1.16)	.745
Brachioplasty	3.13 (1.79-6.02)	<.01	1.85 (1.37-2.50)	<.01	1.53 (1.10-2.15)	.0121	1.19 (0.796-1.84)	.403
Thighplasty	3.51 (2.15-6.17)	<.01	1.51 (1.16-1.95)	<.01	1.25 (0.954-1.65)	.106	1.13 (0.819-1.58)	.465
Liposuction	1.84 (1.57-2.17)	<.01	1.73 (1.48-2.01)	<.01	1.22 (1.03-1.43)	.0204	1.36 (1.15-1.61)	<.01

We performed a subgroup analysis looking at the relationship between preoperative comorbid conditions and postoperative outcomes among the individual procedures (panniculectomy, abdominoplasty, brachioplasty, thighplasty, mastopexy, breast augmentation, augmentation mastopexy, breast reduction, and trunk liposuction) and found that for all procedures, patients with diabetes, hypertension, obesity, and tobacco were significantly more likely to develop a wound dehiscence, hematoma, or a surgical-site infection (Table 5).

## DISCUSSION

This is the largest and most recent study in the literature examining preoperative comorbid conditions and postoperative complications in patients undergoing body contouring surgery. We identified 243,886 patients who underwent body contouring surgery, some of whom had more than 1 type of body contouring procedure, which explains the greater number of counted procedures relative to the number of patients. The analysis revealed baseline characteristics and complications for patients undergoing body contouring surgery.

The most common comorbid conditions in our patient population were hypertension (73,091; 30.0%), obesity (55,051; 22.6%), and diabetes (34,794; 14.3%; Table 3). Based on national statistics, the frequency of patients in the general population with hypertension is 47.0%, obesity is 39.0%, and diabetes is 10.5%.^9^ The rates of surgical-site infection, hematoma, and wound dehiscence within 90 days of having body contouring surgery were 1.8%, 2.5%, and 3.3%, respectively (Table 4). The previously reported rates of surgical-site infection ranged from 1.1% to 15%^10^ and wound complications were reported up to 15%.^11^ Previously reported rates of hematoma in body contouring surgery ranged from 3% to 15%,^12-14^ while major hematoma was reported at 0.91%.^15^

Most patients undergoing body contouring surgery in the study were middle-aged females. Similarly, American Society of Plastic Surgery (ASPS) Plastic Surgery Statistics Report published Cosmetic Plastic Demographic Trends. It reported that patients aged 40-54 make up the majority of cosmetic procedures in the United States, including 684,000 patients who undergo surgical intervention and 5.4 million that undergo minimally invasive procedures. Females make up the vast majority of patients undergoing body contouring surgery, with 92% of all cosmetic procedures or 12.4 million total cosmetic procedures.^1^ Our analysis showed that the majority of cases performed were breast procedures (breast reduction, mastopexy, and breast augmentation; 203,566; 70.1%), followed by abdominal procedures (panniculectomy, abdominoplasty, and liposuction to the abdomen and flanks; *n* = 82,110, 28.3%) and extremity procedures (thighplasty and brachioplasty; *n* = 4,916, 1.7%; Table 1). Our data are similar to the 2020 ASPS Plastic Surgery Statistics Report that breast procedures were the most common surgical procedure (*n* = 157,258), followed by abdominal procedures (*n* = 97,987) and extremity procedures (*n* = 23,722).^1^

Our data showed that patients with diabetes were 1.38 times more likely to develop a surgical-site infection, 1.50 times more likely to develop a wound dehiscence, and 1.39 times more likely to develop a hematoma than patients who do not have diabetes. This is not surprising as diabetes has a complex and significant relationship with wound healing and infection. Wound healing is a dynamic process that diabetes impairs through chronic inflammation, microcirculatory and macrocirculatory dysfunctions, hypoxia, autonomic and sensory neuropathy, and impaired neuropeptide signaling.^16^ A systematic review on diabetes and the risk of surgical-site infection found that diabetes is an independent risk factor for surgical-site infection in multiple surgical procedure types, including breast reconstruction. This is felt to be partially related to hyperglycemia, resulting in increased immune dysfunction (including damage to the neutrophil function, depression of the antioxidant system, and humoral immunity), as well as microangiopathy and macroangiopathy.^16,17^

Additionally, patients with hypertension were 1.45 times more likely to develop a surgical-site infection, 1.60 times more likely to develop a wound dehiscence, and 1.72 times more likely to develop a hematoma. Because the precise mechanism behind the increased rates of surgical-site infections and hematomas has not been fully described, previous studies have also noted delayed wound healing in patients with hypertension.^18^ Certainly, hematoma formation has a negative impact on wound healing, recovery time, and overall morbidity, and as such these complications can be interlinked.^19^

Obesity as a risk factor for postoperative complications is well established in the surgical literature, including in plastic surgery. Our analysis showed that obese patients were 2.23 times more likely to develop a wound infection, 1.89 times more likely to develop a wound dehiscence, and 1.20 times more likely to develop a hematoma. A similar relationship has been established in several studies within cosmetic surgery. Au et al demonstrated a significant increase in the occurrence of complications among morbidly obese and severely morbidly obese patients undergoing a single body contouring procedure, and the risk of complications increases with the worsening degree of obesity.^5^ Likewise, Sinha et al illustrated that obesity is a significant risk factor for surgical-site infection in patients undergoing both implant-based and autologous breast reconstruction.^20^ A meta-analysis by Bigarella et al focused on patients undergoing plastic surgery procedures, including cosmetic procedures and breast reconstruction, found that obese patients were at the highest risk of developing medical or surgical complications.^21^ Obesity has a multifaceted effect on wound healing, including the inherent anatomic features of adipose tissue (avascularity), vascular insufficiencies (impaired angiogenesis), cellular and composition modifications (chronic low-grade inflammation), oxidative stress, alterations in immune mediators (higher levels of pro-inflammatory markers), and nutritional deficiencies.^22^ All of these factors can contribute to the increased prevalence of postoperative complications in obese body contouring patients.

The effects of tobacco use on postoperative surgical complications have been extensively studied, specifically the effects on wound healing. Our study demonstrated that patients who used tobacco were 1.79 times more likely to develop a surgical-site infection, 1.71 times more likely to develop wound dehiscence, and 1.38 times more likely to develop a hematoma. Our findings are concurrent with the established mechanisms of increased wound-healing complications in patients who use tobacco products. Tobacco has a significant impact on wound healing and infection due to vasoconstrictive effects, microvascular injury, impaired oxygen, and cell dysfunction.^23^ In addition, tobacco smoke contains toxins that promote the formation of free radicals, resulting in damage and dysfunction of the vascular endothelium, which can result in rupture of small arterial walls and promote coagulation disorders leading to continuous bleeding.

The data presented in our study are integral for providers performing body contouring procedures, as it identifies patients who are at increased risk of postoperative complications. We recommend a comprehensive history and physical examination of all patients undergoing body contouring procedures and a close partnership with primary care providers to ensure strict glycemic control, blood pressure control, and smoking cessation prior to such procedures. In our practice, we require diabetic patients to have a HgA1c <7% prior to surgery, and smokers to have a negative urine and/or blood cotinine test prior to undergoing body contouring surgery with a commitment to avoid nicotine products for at least 6 weeks before and after surgery. Moreover, it is not uncommon for patients undergoing body contouring procedures to have undergone bariatric surgery for obesity but still have a body mass index >30 kg/m^2^ even after significant weight loss. In this circumstance, the data provide clinicians with quantitative data on a patient's risk of developing several postoperative complications and facilitate an informed discussion with the patient.

There are several advantages to using a large database to evaluate comorbid conditions and complications among patients undergoing body contouring procedures. PearlDiver software is based on insurance claim data, and therefore, they have a vested interest in maintaining accurate records. The database is diverse in that it includes different surgery (ie, office-based surgery suites, accredited surgical centers, and hospital-based settings) across all 50 states in the United States and data collected over several years, allowing the authors to make generalizations about the results and translate them into clinical practice.

Although PearlDiver software is based on insurance data, it contains data about procedures that can be considered cosmetic and not typically covered by insurance plans. It is worth noting that the definition of what constitutes a “cosmetic” procedure can vary depending on the insurance company and the specific policy. For example, some insurers may consider certain procedures to be medically necessary if they alleviate pain or improve function, even if they are also considered cosmetic in nature. Ultimately, the data available in PearlDiver will depend on the terms of the insurance policy and any optional benefits or riders selected by the policyholder.

This study is not without limitations. Given that the PearlDiver software is based on insurance claim data, the data are only as accurate as is allowed by proper CPT coding for the included procedures. The large database does not account for surgeon-specific factors such as surgical techniques that could confound the results. A large number of body contouring procedures are considered aesthetic cases and are not covered by insurance, and therefore, the database may not be capturing a sufficient number of cases to make generalizations. Comorbid conditions and complications are based on ICD-10 codes, making it difficult to discern patient-specific details. For example, we can capture patients who are smokers but are unable to discern the number of cigarettes smoked per day, or if there was temporary smoking cessation prior to surgery. The database does not differentiate between small complications that can be treated with outpatient management vs clinically significant complications requiring return to the operating room. We did not look at complications past 90 days and may have underreported complications. Since this study was directed at comorbid conditions and complications in body contouring procedures, further investigation is required to compare bariatric and nonbariatric patients. Bariatric patients develop a unique profile of deficiencies secondary to massive weight loss, such as vitamin, mineral, and protein calorie malnutrition—predisposing them to complications.

## CONCLUSIONS

Our data suggest that patients with obesity, tobacco use, diabetes, and hypertension undergoing body contouring surgery are at greater risk of developing wound dehiscence, hematomas, and surgical-site infections. Understanding this data is imperative for providers to adequately identify associated risk factors, stratify patients, and provide adequate counseling. In their impactful role in patient's lives, surgeons have a duty to look beyond their actions in the operating room and be cognizant of the predisposing factors to postoperative complications. By providing a comprehensive investigation of the abundance of comorbid conditions and their effects on postoperative complications, this information can help guide surgeons in their procedural decision-making and in obtaining appropriate informed consent for patients interested in undergoing various body contouring procedures.
